# Value and Implementation of Patient and Public Involvement and Engagement in Health Technology Assessment for Japan: implications from systematic searches

**DOI:** 10.1017/S026646232500008X

**Published:** 2025-02-28

**Authors:** Takako Kaneyasu

**Affiliations:** Department of Biomedical Sciences, College of Life Sciences, Ritsumeikan University, Kusatsu, Shiga, Japan

**Keywords:** patient and public involvement, patient engagement, public engagement, health technology assessment

## Abstract

**Objectives:**

This study comprehensively reviewed reports on patient and public involvement and engagement (PPIE) in health technology assessment (HTA) overseas and identified the status and possible future measures, of PPIE in Japanese HTA.

**Methods:**

The series of reviews targeted systematic reviews (SR#1), references in SR#1 (SR#2), and new articles after SR#1 (SR#3). The MEDLINE and Embase databases were searched through August 2024 using the terms “patient involvement/engagement,” “patient participation,” “community participation,” “public involvement/engagement,” and “health technology assessment.” The implementation details were extracted from information published on the websites of the HTA agencies.

**Results:**

Three review articles in SR#1, 12 references in SR#2, and 10 articles in SR#3 were selected. The opportunities for countries, including Japan, to participate in discussions on the HTA process did not differ significantly; however, information on PPIE in Japan was scarce and did not indicate their purpose and value.

**Conclusions:**

Collected articles indicated that the value of PPIE in HTA includes relevance, equity, fairness, legitimacy, and capacity building. The participation of patient and public representatives in Japanese discussions since 2005 appeared to be very limited to consider PPIE in HTA. In countries that implement PPIE in HTA, the value of PPIE is explicit: the process guidelines are specific and provide an appropriate environment for input that includes education, training, and feedback. Future reforms of the Japanese system will require discussions on PPIE purpose and value, implementation, and creating an environment in which a diverse range of patients and the public can easily express their views.

## Background

Health technology assessment (HTA) schemes were first employed in the United States (1;2) and have been institutionalized in Australia and Canada (1;3;4). After its institutionalization in the United Kingdom (5), HTA has been introduced in various countries in Europe and the Asian region (1). Patient and public involvement and engagement (PPIE) has been implemented in clinical research (6;7), clinical practice guideline development (7;8), and decision-making in various areas of healthcare, with PPIE in HTA taking a variety of forms (1;2;9).

In Japan, pharmaceuticals and medical devices approved by the regulatory authority have been covered by public healthcare insurance without consideration of cost-effectiveness for a long time. The prices are set based on predetermined rules, with proposed prices that have been agreed upon in discussions between companies and the Ministry of Health, Labour, and Welfare (MHLW) and then authorized by the Central Social Insurance Medical Council (Chuikyo) (10,11). A system for the evaluation of the cost-effectiveness of healthcare technologies (Japanese HTA) was introduced in 2019. Japanese HTA is designed to complement the existing system (that is on, to adjust the price once it has been decided using the results of cost-effectiveness evaluations), and the products and price adjustment ranges of the determined price, and they are finally approved by the General Assembly in Chuikyo (12–14). Patient and citizen representatives have been participating in various committees in Chuikyo since the 2005 reform of the system in response to the bribery scandal (15–17). These representatives participate as members of the Expert Committee on Cost-Effectiveness Evaluation (CEE) (Expert Committee of CEE), which deliberates on individual items; the Special Committee of CEE (Special Committee of CEE), which examines the system’s form; and the General Assembly, which approves discussions at Expert Committee and Special Committee of CEEs. (18,19).

*Currently, Chuikyo mainly comprises three parties: the payer, who bears the cost of medical care; the medical practitioner, who provides medical care; and the public interest, which coordinates between the two. The General Assembly of Chuikyo includes seven members representing the insured (including patient harmed by an adverse drug or medical accident and citizen/worker representatives), insurers, and employers (payer); seven members representing medical practitioners (including five doctors, dentists, and pharmacists); six members specializing in law, politics, economics, and so forth (public interest); and four expert members from medical professionals not included in the medical practitioners. The personnel structure of the Special Committee of CEE consists of six representatives of the payer (including patient and citizen representatives), six representatives of the medical practitioners*, *four public interest representatives, and four pharmaceutical and medical device industry representatives. The personnel of the Expert Committee of CEE include experts in medical economics, clinical medicine, medical statistics, medical ethics and law, as well as patient and citizen representatives (17*–*19).*

However, the Japanese HTA system is in its infancy, and available information is fragmented, making it difficult to understand how PPIE is being implemented. Owing to the deteriorating fiscal situation in Japan, an expanded scope of the HTA is currently being considered (20), and the value of the PPIE in the HTA system is expected to increase.

PPIE in HTA agencies has been examined from the perspectives of the value of involvement (21–23), methods of involvement (2; 21;24), and resources for involvement (25). The present review synthesizes the findings from existing studies and several country agency documents on PPIE in HTA to confirm PPIE value, implementation, and need and identify measures needed for the future of PPIE in Japan.

## Objective

By organizing the value, implementation methods, and necessary resources for PPIE in HTA, this study will clarify the current state of PPIE in the Japanese HTA system and identify the measures and resources required to improve the system.

## Methods

Information on PPIE value, implementation, and required resources was extracted through umbrella and subsequent reviews. Details of the implementation, which were difficult to ascertain from the literature, were extracted in a predefined format from the public information available from the websites of HTA agencies. The differences between Japan and other countries were examined by adding Japanese information to the extracted data.

### Umbrella and scoping reviews

#### Search method

***Umbrella review***: The MEDLINE and Embase databases were systematically searched to identify systematic reviews published up to 31 August 2024. The search terms were: “patient participation,” “patient involvement,” “patient engagement,” “community participation,” “public involvement,” “public engagement,” and “health technology assessment.”

***Scoping review***: A complementary literature search of the MEDLINE and Embase databases was also performed to identify original articles or documents published after the umbrella review (between 1 January 2020 and 31 August 2024). The search terms were “patient participation,” “patient involvement,” “patient engagement,” “community participation,” “public involvement,” “public engagement,” “technology assessment,” “the Pharmaceutical Benefits Advisory Committee (PBAC),” “the Canadian Agency for Drugs and Technologies in Health (CADTH),” and “the National Institute for Health and Clinical Excellence (NICE).” As 96 percent of the articles from the umbrella review search yielded not-HTA-eligible articles, the scoping review employed “technology assessment” as a single phrase in the search, supplemented by abbreviations for the three target agencies.

#### Article selection

Articles on PPIE in the HTA that met the following criteria were included:

***Umbrella review***: The included articles were required to be reviews of original articles published from countries belonging to the Organization for Economic Cooperation and Development or in Asia. Study designs, including guidelines, qualitative studies, case studies, and cross-sectional surveys, were excluded.

***Scoping review***: The included articles were required to be original articles or documents (that is on guidelines, qualitative studies, case studies, and cross-sectional surveys) published in Australia, the United Kingdom, or Canada.

The exclusion criteria were nontargeted outcomes, including PPIE on patient-reported outcomes (patient preferences and patient experiences), PPIE on medical procedures not covered by the Japanese HTA system (that is on vaccinations, tests, fertility treatments), or PPIE focused on ethical issues, such as efficiency and equity of healthcare; local-level HTA; publication style (that is on editorial, commentary, and discussion); and scope (prioritization not considered in the Japanese HTA).

The umbrella review included reviews obtained directly from the database (primary screening articles) and the references of the selected reviews. The references (secondary screening articles) were reviewed using the same criteria as in the scoping review and added to the article set.

#### Data extraction and integration

The following contents were extracted from the articles from the umbrella (primary and secondary screening) and scoping reviews: (i) author, (ii) publication year, (iii) country, (iv) study type, (v) main theme, (vi) participants (patient or public), (vii) PPIE values, (viii) PPIE implementation (target HTA stages and participation type), and (ix) resources (guidelines, education and training, information sharing or dissemination from agencies, actions for patients and public inputs, and feedback or process evaluation). Items (i)–(vi) were mandatory, while (vii)–(ix) were collected if available.

### Website search

#### Search methods

The search target was information from the websites of three HTA agencies: PBAC in Australia, CADTH in Canada, and NICE in the United Kingdom, as well as the Centre for Health Economics and Evaluation (C2H) of the National Institute of Health and Medical Sciences in Japan as documents on the evaluation/deliberation process on the website of Chuikyo in Japan. This information was updated on 31 August 2024.

#### Information integration

The descriptions of PPIE in the assessment/deliberation process were consolidated into the following items based on previous articles (2;21): institution name, role (recommendation/resolution), PPIE participants, PPIE-related statement, PPIE for conducting HTA; PPIE on review assessment results and the formulation of recommendations (or reports for drug price adjustments in Japan); PPIE in the dissemination of decision or assessment results; and internal evaluation/feedback.

In the United Kingdom, NICE reviews and formulates recommendations and makes decisions on reimbursement. In Australia and Canada, the roles of HTA agencies (PBAC and CADTH) are limited to reviewing and formulating recommendations, with decisions being made by a separate agency (Table 2). As the drugs to be evaluated are selected in Japan based on predefined selection criteria (13), PPIE information from the subsequent process was collected and integrated in this review.

## Results

The search for the umbrella review identified 3,220 candidate articles, from which three reviews were finally extracted (primary screening). A total of 54 references were obtained from the three extracted reviews. Most of these 54 references referred to information from Australia, Canada, and the United Kingdom. Therefore, the scope was narrowed to articles from these three countries, and 12 articles were included in the review (secondary screening).

**Figure 1.**
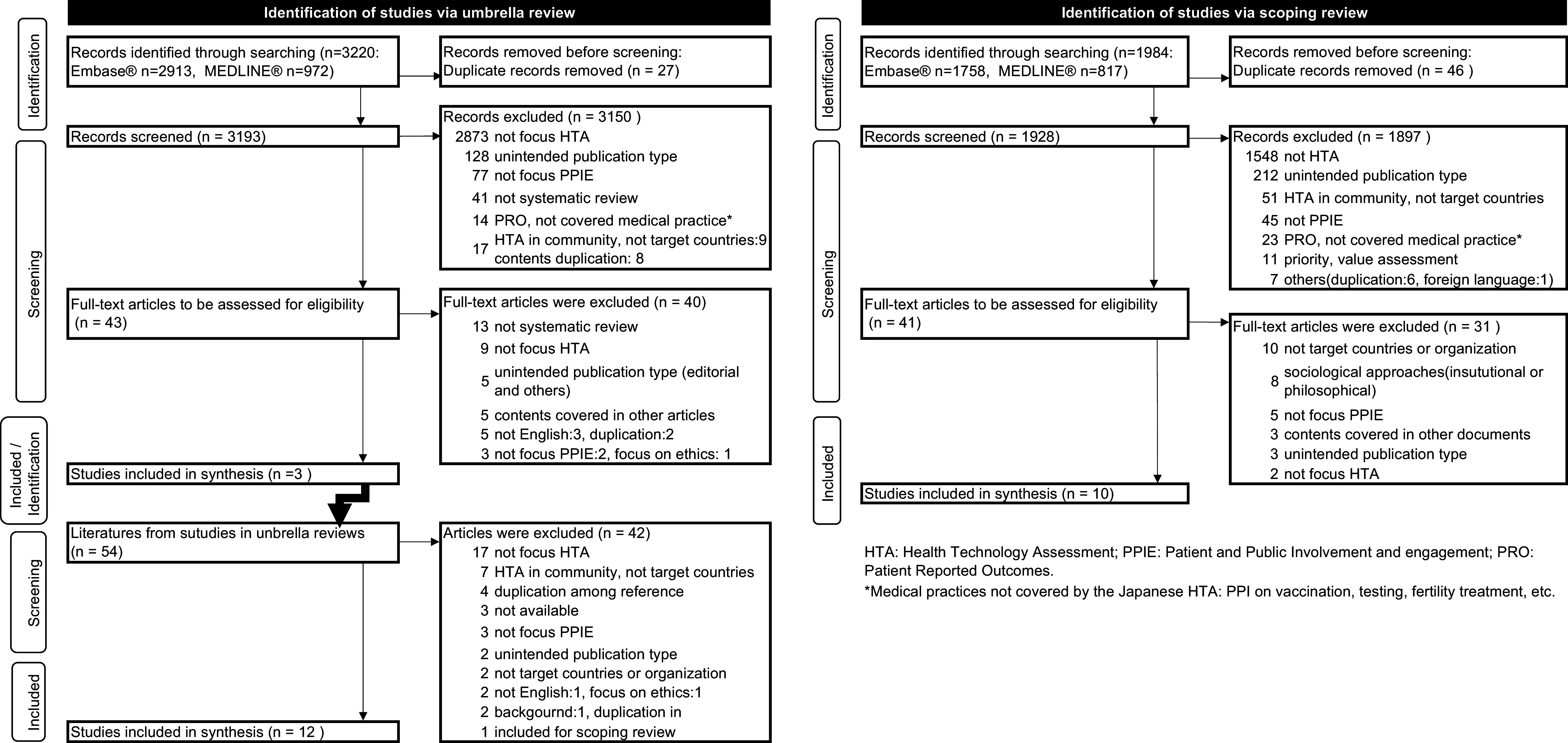
PRISMA flow diagram. PRISMA, Preferred Reporting Items for Systematic reviews and Meta-Analyses.

The scoping review targeted articles from three agencies in Australia (PBAC), Canada (CADTH), and the United Kingdom (NICE) from 2020 onward because Gagnon et al. (25), which was selected in the umbrella review, comprehensively reviewed reports up to 2020. The search for scoping reviews identified 1,984 candidate articles; however, among these, only 10 articles were included. The sequences of these searches are presented in the Preferred Reporting Items for Systematic Reviews and Meta-Analyses (PRISMA) chart in Figure 1.

The articles selected for the series of reviews included qualitative studies (9 articles) and others (16 articles), with the latter comprising both multicountry studies (five articles in total), including reviews and guidance development, and single-country studies (11 articles in total). Table 1 provides an overview of the five articles on multicountry reviews and guidance development. Reviews from single countries and qualitative studies are outlined in the Supplementary Appendix. Information on the HTA process and PPIE in different countries is presented in Table 2 and 3.

**Table 1.** Outline of the multinational literature review

Author (year)	Study type	Main theme	Participants	PPIE purpose/value	PPIE implementation	
					Stage of HTA	Form of involvement
Pinho-Gomes (2022) (21)	Scoping review	Summarize PPI principles, values, frameworks, and strategies in HTA and guideline development to evaluate the impact of PPI	Patients and public	Representation, transparency, relevance, equity, fairness, and reconciling different types of knowledge	Selecting and prioritizing topics, scoping, evidence review and analysis, drafting recommendations, and PPI dissemination and evaluation	Individual or collective level: communication, consultation, and participation
Gagnon (2021) (25)	Systematic review	Synthesize the barriers and facilitators of PPI in HTA and propose a framework to assess its impact	Patients and public	Raise awareness, relevance of PPI, perceptions of other stakeholders, develop patient materials, and identify collaborators	Different and each stage of HTA	Group or individual patients: participation, consultation, or indirect participation
Mason (2020) (22)	Scoping review	Overview of methods for the evaluation of patient involvement impact	Patients	Patient involvement provides insights into the technology under review that was not otherwise available。	From scoping topics, to interpreting evidence, and even drafting recommendations	Patient group: direct involvement on HTA committees, and multiple forms
Hunter (2018) (27)	Guidance document	Provide recommendations for activities to support patient involvement in HTA bodies and specific guidance for individual HTA processes	Patients	Relevance, fairness, equity, legitimacy, and capacity building	Identifying and prioritizing. Scoping, assessing, reviewing and disseminating	Group or individual: communication, consultation, and participation/written submissions
Oortwijn (2022) (33)	Good practices report	Guidance and checklist development for deliberative processes for HTA	Patients, public/citizens, and other stakeholders	Process indicators (e.g., transparency, impartiality, inclusivity, timeliness consistency, and verifiability) and outcome indicators (e.g., increased public trust, and acceptance of decisions)	Identification and prioritization of relevant topics for HTA, providing scientific advice, scoping, assessment, and synthesis of relevant information, contextualization of HTA, development and communication of the output(s), monitoring and evaluation	No info about group or individual: Face-to-face, virtual, written

Author (year)	Resource Guidelines	Education/training	Info from institutions	Patient/public input	Evaluation/feedback
Pinho-Gomes (2022) (21)	Abelson (2016), EUPATI (2018), RedETS(2019), Perfetto (2018), CADTH (2021), HTAi (2022), INAHTA (2021)	de Wit (2019), CADTH (2021) & EUPATI: (2018) Support/training for patients and orgs Expertise and training for staff	Gagnon (2015), Toledo - Chavarri (2019), Hunter (2018)	de Wit (2019), CADTH (2021) & EUPATI: (2018)	de Wit (2019), CADTH & EUPATI: PPI feedback, review, and evaluation
Gagnon (2021) (25)	GRIPP checklist (2011/2017)	Organizational context: i.e., providing documents and training for patients in advance with adequate materials	Raise awareness, i.e., better information from government institutions to patients and public about HTA	Organizational culture: demonstrating openness toward patients’ perspectives	Decision-making context: providing feedback about their participation
Mason (2020) (22)	n/a	n/a	n/a	n/a	Combination of both qualitative and quantitative strategies may allow for comprehensive assessment
Hunter (2018) (27)	HTAi/ISPOR materials, EUnetHTA Core Model, EUPATI guidance materials	Patients: mentoring and training about HTA. Other HTA participants: training about involvement and consideration of patients’ perspectives	Provide lay language versions of HTA outcome documents	Make systems for written submissions easy to use and appropriate support for individuals making submissions	Regular reflection and review Feedback to patients: how their submissions inform specific HTA
Oortwijn (2022) (33)	Jansen (2018), HTAi patient group submission template (2014), Good Practices Report of a Joint HTAi/ISPOR Task Force (2022)	Training and education should be considered so that individuals can participate fully in an informed deliberation	Recording (video, audio, or transcript), written report (e.g., minutes)	Manage power dynamics that can lead to distractions and dilute fruitful discussion	Monitoring and evaluating: a deliberative process involving questionnaires, interviews, document reviews, or live meetings

Abbreviations: CADTH: Canadian Agency for Drugs and Technologies in Health, EUnetHTA: European Network for Health Technology Assessment, EUPATI: European Patients’ Academy on Therapeutic Innovation, GRIPP: Guidance for Reporting Involvement of Patients and the Public, HTA: health technology assessment, HTAi: HTA international, INAHTA: International Network of Agencies for Health Technology Assessment, ISPOR: International Society for Pharmacoeconomics and Outcomes Research, n/a: not applicable, orgs: organizations, PPI: patient and public involvement, RedETS: Spanish Network of Health Technology Assessment

**Table 2.** Outline of the Web search for information on PPIE in HTA in four countries

Country	HTA institution	Role	Participants as PPIE	Policy/statement
Australia	Pharmaceutical Benefits Advisory Committee (PBAC)	Review and recommendation	Organizations and individual consumers	Enhanced Consumer Engagement for HTA
	Department of Health and Aged Care	Price negotiation	n/a	n/a
Canada	Canadian Agency for Drugs and Technologies in Health, Common Drug Review (CADTH)	Review	Patients and the general public	CADTH Framework for Patient Engagement in Health Technology Assessment
	CADTH Expert Committees	Recommendation	Public	n/a
	Provincial/territorial pharmaceutical benefit plans	Decision	n/a	n/a
United Kingdom	National Institute for Health and Cera Excellence (NICE) /Technology Appraisals Committee	Review, recommendation, and decision	Lay members (patients, carers, and so forth, and members of the public)	Patient and public involvement policy
Japan	Center for Outcomes Research and Economic Evaluation for Health (C2H)	Review	n/a	n/a
	Expert committee at Chuikyo	Recommendation for drug price adjustments	Public (academic expert*), patient expert	n/a
	General meeting at Central Social Insurance Medical Council (Chuikyo)	Decision regarding drug price adjustments	Public (academic expert*), one citizen** and a patient group representative	n/a

Abbreviations: HTA: health technology assessment, n/a: not applicable, PPIE: patient and public involvement and engagement.

* Academics representing the public interest participate as individuals. They are involved in the adjudication and therefore do not comment on other occasions as a rule in Japan.

** Citizen is a representative of a labor organization.

**Table 3.** Outline of the Web search on PPIE at each stage of HTA

	Country			Australia		Canada	United Kingdom	Japan	
	Institution			PBAC		CADTH	NICE	Expert committee at Chuikyo	General Assembly at Chuikyo
Stage of HTA implementation	Conduct HTA	Scoping (pre-submission meeting)	Consultation/workshop/comments and discussion	Comment/consultation: consumer organizations		Written comments for anticancer drugs, not on the rapid algorithm: patient group	Consultation/workshop: patient organizations/ lay members	Comments on the discussion: (public)/patient expert	n/a
		Company report (submission)	Hearing/comments	Hearing: organizations and individual consumers		Written comments: patient group or individual	Written comments: patient organizations/lay members, patient expert	Comments on the discussion: (public)/patient expert	n/a
		HTA results (draft report)		n/a		n/a	n/a	n/a	n/a
	Review of HTA results and formulation of recommendations /reports	Committee meeting (consultation)	Discussion (comments)	Organizations and individual consumers with the disease or condition	Consumer representative, who reviews and collates the comments for the PBAC agenda	Public with a lay perspective, (Patient group comments provided as ‘patient input’)	Comments on the discussion: lay members, patient expert, patient organization	Comments on the discussion: (public)/patientexpert	n/a
		Final guidance	Comments/appeal	Appeal: when applicants are patients (otherwise n/a)		Comments/feedback: patient group	Comments/appeal: patient organizations, patient experts	Comments: (public)/patient expert	Comments: (public) citizen and patient organizations
Post HTA	Dissemination of decision/HTA findings	Publication	Plain language summary	n/a		n/a	Lay member: Information for the Public	n/a	n/a
	Internal evaluation/feedback			n/a		Procedural review, Feedback letters to patient groups (Berglas 2016)	Lay member: exit survey	n/a	n/a

Abbreviations: C2H: Center for Health Economics and Evaluation of the National Institute of Health and Medical Sciences; CADTH: Canadian Agency for Drugs and Technologies in Health; Chuikyo: Central Social Insurance Medical Council; HTA: health technology assessment; n/a: not applicable; NICE: National Institute for Health and Clinical Excellence, PPIE: patient and public involvement and engagement.

The following section describes the findings from the information obtained on PPIE value, implementation, and required resources.

PPIE value.

Based on research reports by patient groups (26), the value of patient involvement is relevance, equity, fairness, legitimacy, and capacity building (27). HTA agencies have reported that PPIE ensures legitimacy and fairness in resource allocation decisions (28) and provides a background to the technology under evaluation (29). Pinho-Gomes et al. summarized the value of PPI in representation, transparency, relevance, equity, fairness, and reconciling different types of knowledge. (21). These PPIE values are posted as policies or statements on the website of each agency in Australia (30), Canada (31), and the United Kingdom (32). In contrast, Chuikyo’s website in Japan lacks such statements, and it was not until 2021 that PPIE-related comments were confirmed in the minutes of the meetings.

PPIE implementation (target stage/form of participation):

The results of this review showed that all stages of HTA are subject to PPIE. More specifically, this includes technology identification and prioritization, scoping, evaluation by companies, review by HTA agencies, dissemination of decision or HTA findings, and evaluation of the PPIE process (21;22;25;26;33;34). The details are in Table 2. Guidelines and reports from HTA agencies in countries other than Japan confirm the involvement of patients and the public in each process.

Gagnon et al. (25) broadly classified the forms of involvement as (i) direct participation of patients and the public in decision-making processes (that is on meetings) and (ii) provision of patient and public perspectives through documents (that is on indirect participation). The unit of participation (individual or collective) and the collection of views vary depending on the agency; however, both individuals and groups generally can express their views (25;33;34). Under the Japanese HTA, the views of patients and the public can be reflected in meetings by representatives of patients and labor organizations. Academia, who represents the public interest, participates as an individual and is responsible for coordinating between the payer and the medical practitioner; thus, their opinions on such coordination are limited to what is necessary. (15;16). Only a few mentions regarding PPI were identified in the minutes.

Resources required for PPIE.

The resources required to implement PPIE are time, material, human, and financial (25;34). Based on the feasibility in Japan, this review summarizes the resources needed, including guidelines, education and training, information sharing and dissemination from facilities, information from patients and the public, and process evaluation.

(a) Guidelines.

Many of the articles included in the present review referred to PPIE in the HTA process guidelines of Australia (35), Canada (36;37), and the United Kingdom (38;39), or guidelines by the European Patients’ Academy on Therapeutic Innovation (EUPATI) (27), the European Network for Health Technology Assessment (EUnetHTA) (40), the International Society for Pharmacoeconomics and Outcomes Research (ISPOR), and Health Technology Assessment International (HTAi) (33). However, Japan has no such guidelines on the process itself nor the PPIE, and is limited to addressing technical issues of evaluation.

(b) Education/training.

Low et al. (41) comprehensively summarized information regarding education/training programs in PPIE implementation provided by the EUPATI, ISPOR, HTAi, and HTA agencies. The authors reported that accessibility, inclusiveness, transparency, and interpersonal relationships and committee dynamics (33;42) required consideration and that tailored support was important (27;43;44). Moreover, education and training is also needed for participants, including researchers, staff, HTA reviewers, and committee members (21;27). No information on education/training by relevant Japanese institutions could be found.

(c) Information from institutions.

Much of the information on the implementation of HTA in countries implementing PPIE is shared collectively (45). In addition, simplified versions are also provided when disseminating the evaluation results to the public (27;34). Through this process, PPIE is considered to contribute to promoting the understanding of the HTA system (25). In Japan, relevant information is provided separately by the three meetings of the Chuikyo (that is the General Assembly (46), the Expert Committee of CEE (47), and the Special Committee of CEE (48) and by the C2H (49–51), which compiles official analysis reports. However, only some of the minutes of the Expert Committee CEE meetings have been made public, and no lay summaries have been seen for the reports.

(d) Patient and public input.

A range of opportunities and support should be available to provide information to patients and the public. Regarding patient and public responses, guidances (25;27;41) on information for patients and the public (Summary of Information for Patients) and templates for patient input (52–54) are provided. However, collating the collected opinions and using them in discussions and reports remain a challenge, even in other countries (21;26;55–57). As noted in ‘PPIE implementation’, the relevant institutions in Japan do not have a mechanism for receiving input directly from patients and the public specifically regarding the evaluation of cost-effectiveness.

(e) Process evaluation and feedback.

The PPIE process can be evaluated through qualitative research, such as interviews (21;25;33;34;55), quantitative evaluations, such as questionnaires (22;26;29;33), and reports. The research is conducted by external evaluators (22;43;55–57) and HTA agencies themselves (39;58) and some provide feedback (59).

## Discussion

Based on the reports of PPIE in HTA in other countries, the current PPIE in Japan and the measures and resources needed to improve it are described below.

PPIE purpose and value.

PPIE values in HTA generally include relevance, equity, legitimacy, and capacity building, although these differ between countries (21;27). The websites of HTA agencies other than that in Japan consolidated information on PPIE and indicated its purpose and value. Although patients and citizens participate in Chuikyo meetings in Japan, no information on PPIE is provided in the related websites.

Three years after the introduction of HTA in Japan in 2019, a reference to patient involvement was finally confirmed in the minutes of Special Committee of CEE regarding the revision of the system in 2021 (60). In that meeting, patient and citizen representatives questioned whether there was a possibility of future involvement in the form of patients providing input for appraisals, as are done in other countries. The secretariat of the Committee replied, “the purpose of patient participation had not been specifically discussed in the committee before and that it was an issue for the future.” In response, patient and citizen representatives indicated that the background of patient involvement and engagement included factors of consideration for “patient discrimination and people with disabilities.” During the final stages of institutionalization, various issues were discussed regarding “how to deal with factors other than cost-effectiveness” (61), however, PPIE did not appear in the discussions.

Since the 2005 reform, patient and citizen representatives have participated in discussions at Chuikyo to reflect the voices of patients and citizens (15). These patients are participating from the payer’s side, or rather, from a position closer to the public. This differ from position of PPI patients in HTA in other countries, but the form of participation was maintained, without discussion, in the creation of the Japanese HTA system. However, as the PPIE value is diverse and meant to ensure the legitimacy of decisions, a discussion on the value and implementation of PPIE in future revisions of the Japanese HTA is inevitable. Previous revisions of the Japanese system have discussed the training of experts to perform technical assessments (60,62), but future expansions of the system should also discuss the training of experts to implement PPIE.

PPIE implementation.

HTA in the three countries other than Japan clearly describe PPIE in the guidelines and reports and include a variety of participation units and opportunities for participation. In contrast, the Japanese HTA lacks process guidelines, and the participation of patient and citizen representatives in the three meetings (General Assembly, Expert Committee of CEE, and Special Committee of CEE) must be confirmed in the respective meeting rosters and minutes (18;19;47;48). The participation in these meetings includes representatives of the public interest, labor organizations, and patient groups, or patient experts with disease-specific expertise as well as technical knowledge of pharmaceutical research and development and regulatory affairs through experience in drug litigation (1 8;1 9).

While only a few mentions of patient and citizen representatives were identified, the minutes recorded a request to establish a system that would enable them to listen to the opinions of a wide range of patient groups and to evaluate them comprehensively (60). This suggests that the representatives have the opportunity to participate in the meetings, but that participants lack sufficient resources to express their opinions. Opportunities are lacking for patients and citizens to express their views directly outside of meetings and no mechanism exists to encourage the expression of these opinions. Even if people struggle to express their opinions, no clear mechanism exists to link these opinions to measures or to provide feedback to patients and citizens. Thus, guidelines on processes, including PPIE, are needed, and patients and citizens should conduct process evaluations.

Resources required for PPIE.

In countries studied other than Japan, patients and public representatives are involved in the discussion at each stage of HTA evaluation, and environments have been developed to provide multiple methods for input to be provided. The resources required to develop these environments included process guidelines, education and training, information sharing and provision from institutions, and various opportunities and support systems for input and feedback.

As mentioned above, Japan lacks HTA process guidelines and information about education and training. PPIE information in clinical research is provided by patients and public groups (Japanese translation of EUPATI’s PPI-related educational program (63;64). Moreover, PPIE information in clinical practice guidelines (8) includes a section on HTA, which is useful for understanding the situations in other countries. However, the Japanese HTA differs significantly from other systems in terms of reimbursement and scope of covered technologies. Promoting an understanding of the Japanese HTA requires explanatory information from C2H (49) and lay (plain language) summaries of various reports on the discussions at Expert Committee of CEE (50,51).

Education and training are also necessary for those who accept to participate. Additionally, the application of information obtained through PPIE will enhance the value of PPIE.

Many HTA agencies in this review have centralized and provided information on evaluation findings (45). In Japan, relevant information is provided separately by the three meetings of the Chuikyo (46–48) and by the C2H (49–51), making it difficult for the public and patients to obtain comprehensive information and judge the content of such reports. As discussed above, a variety of input opportunities for participants is required, as well as a support system to provide feedback to participants and set up contact points for individual consultations.

Patient and citizen participation in the current Japanese HTA follows the structure of Chuikyo, which was reformed in 2005 and is very limited in terms of being considered PPIE in HTA. Although PPIE purpose, value, and implementation has not been discussed and the resources (education/training and information sharing) required to comment on the discussions are not sufficient, patient and citizen representatives have expressed their views in these discussions. However, whether their comments can lead to measures is unclear, and no mechanism is available for patients and citizens to receive feedback on subsequent responses or to evaluate the outcomes of their participation.

PPIE value and implementation must be specifically considered in discussions of future revisions. For this purpose, resources must be secured for patients and the public toward discussion. Patient and public access to HTA information, and discussion of PPIE in HTA outside the system should be increased.

## Limitations

The present study has some limitations. The first is the choice of keywords in the literature search, particularly in the scoping review, which forced the use of “technology assessment” as a single phrase. However, a preliminary review confirmed that the review is broader than the single phrase “health technology assessment” and that the addition of the abbreviations of the HTA bodies (NICE, CADTH, and PBAC) prevents omissions. These measures were necessary to ensure more efficient and accurate reviews. Second, the selection of search targets excluded reports on PPIE with a focus on ethics. However, reviewing reports on ethics requires a different approach (that is on qualitative synthesis methods); thus, future reviews by experts in this area are needed. Third, the search for websites was limited to three HTA organizations. Future reviews with more collaborators will broaden the scope of this survey. Fourth, the granularity of information differed between reports obtained through the umbrella and scoping reviews. The present review complements these differences by citing systematic reviews for matters in which multiple-country cases were considered and citing individual articles for other matters. Fifth is that this review did not perform prioritization as the Japanese system automatically determines which drugs or medical devices are eligible for HTA, based on the price determined in the previous stage of the evaluation process (13). Future expansion of the Japanese system to include reimbursement eligibility will require additional review that includes prioritization. Finally, from spring 2024, Canada’s reorganization from the CADTH to Canada’s Drug Agency and Australia’s comprehensive consideration of HTA process evaluation is a common issue for non-HTA decision-making (65). As of July 2024, these changes have not been identified; however, the possibility of future changes should be noted. In Japan, “patient and public involvement” must be discussed as a common theme for future policymaking in the MHLW (66).

## Conclusion

PPIE in HTA aims to provide patient and public insight into decision-making and has the value of relevance, equity, fairness, legitimacy, and capacity building. In other countries, patient and public representatives are involved in the discussion at each stage of HTA evaluation and mechanisms have been established to allow these representatives to provide input in a variety of ways.

Patient and citizen participation in the current Japanese HTA follows the previous structure, which is very limited when considered as PPIE. Considering the diverse values of PPI, when reforming the system (expanding the scope of coverage), PPIE value and implementation should be discussed and an environment should be created in which diverse patients and citizens can easily express their views.

## Supporting information

Kaneyasu supplementary material Kaneyasu supplementary material

## Supplementary material

The supplementary material for this article can be found at http://doi.org/10.1017/S026646232500008X.

## References

[1] Facey KM, Ploug HH, Single ANV. Patient involvement in health technology assessment. Singapore: Springer. 2017. [cited 2024 Aug 31]. Available from: https://link.springer.com/book/10.1007/978-981-10-4068-9.

[2] Menon D, Stafinski T. Role of patient and public participation in health technology assessment and coverage decisions. Expert Rev Pharmacoecon. Outcomes Res. 2011;11(1):75–89. 21351860 10.1586/erp.10.82

[3] MacPhail E, Shea B. An Inside Look at the Early History of the CADTH Common Drug Review in Canada. 2017. [cited 2024 Aug 31]. Available from: https://www.cadth.ca/sites/default/files/pdf/early_history_of_CDR.pdf.

[4] Hailey D. The history of health technology assessment in Australia. Int J Technol Assess Health Care. 2009; 25:61–67. 19500436 10.1017/S0266462309090436

[5] Raftery J, Powell J. Health technology assessment in the United Kingdom. Lancet 2013; 382: 1278–1285. 24120204 10.1016/S0140-6736(13)61724-9

[6] AMED Research Committee on Survey of Trends in Patient and the Public Involvement in Clinical Research. Patient and Public Involvement (PPI) Guidebook. 2019/03/31. [cited 2024 Oct 31]. Available from: https://www.amed.go.jp/en/ppi/guidebook.html

[7] de Wit M, Cooper C, Tugwell P, et al. Practical guidance for engaging patients in health research, treatment guidelines and regulatory processes: results of an expert group meeting organized by the World Health Organization (WHO) and the European society for clinical and economic aspects of Osteoporosis, Osteoarthritis and Musculoskeletal diseases (ESCEO). Aging Clin Exp Res. 2019;31(7):905–915. 30993659 10.1007/s40520-019-01193-8 PMC6589151

[8] Japan Council for Quality Health Care. How to create a clinical practice guideline, Patient and Public Involvement, Implementation of Patient and Public Involvement. 2023/12/26. [cited 2024 Aug 31]. Available from: https://minds.jcqhc.or.jp/methods/guideline-ppi/practice/.

[9] Stafinskib T, Menon D, Davis C, McCabe C. Role of centralized review processes for making reimbursement decisions on new health technologies in Europe. Clinicoecon Outcomes Res. 2011; 3:117–186. 22046102 10.2147/CEOR.S14407 PMC3202480

[10] Shiroiwa T, Fukuda T, Ikeda S, Takura T. New decision-making processes for the pricing of health technologies in Japan: The FY 2016/2017 pilot phase for the introduction of economic evaluations. Health Policy. 2017;121(8):836–841. 28687183 10.1016/j.healthpol.2017.06.001

[11] Tanaka S. A brief history of the price list for pharmaceuticals covered by Japan’s National Health Insurance system. J. Law Polit. 57 (1), 161–194 (in Japanese).

[12] Hasegawa M, Komoto S, Shiroiwa T, Fukuda T. Formal Implementation of cost-effectiveness evaluations in Japan: A unique health technology assessment system. Value Health. 2020;23(1):43–51. 31952673 10.1016/j.jval.2019.10.005

[13] Fukuda T, Shiroiwa T. Cost effectiveness evaluation of health care technologies in Japan: the New HTA system and the role of C2H. J. Natl. Inst. Public Health. 2021;70 (1) 22–22.

[14] Medical Division. Health Insurance Bureau, Ministry of Health, Labour and Welfare. 22. *Summary of Medical Fee Revision for FY2024* [22. Cost- Effectiveness Evaluation System] March 5, 2024, Edition (in Japanese). 2024/March/5. [cited 2024 Oct 31]. Available from: https://www.mhlw.go.jp/stf/seisakunitsuite/bunya/0000196352_00012.html

[15] Council of Experts on the future of Chuikyo. For a new start for the Central. Social Insurance Medical Council. (in Japanese) 2005/July/20. [cited 2024 Aug 31]. Available from: https://www8.cao.go.jp/kisei-kaikaku/old/minutes/wg/2005/0913/item050913_03.pdf.

[16] Medical Division, Health Insurance Bureau, Ministry of Health, Labour and Welfare. Change in committee member nominations by labor organizations, All Member Roundtable Meeting in the Central Social Insurance Medical Council. (in Japanese) 2004/October/20, 2005/April/6. [cited 2024 Oct. 31] Available from: https://www.mhlw.go.jp/stf/shingi/shingi-chuo_128156.html.

[17] Medical Division, Health Insurance Bureau, Ministry of Health, Labour and Welfare. List of Members of ’the Expert Committee of Cost-Effectiveness Evaluation’ in the Central Social Insurance Medical Council (in Japanese). 2022/April/22 [cited 2024 Aug 31]. Available from: https://www.mhlw.go.jp/stf/seisakunitsuite/bunya/0000121431_00302.html

[18] Law to Amend the Social Insurance Medical Council Act (Act No. 47 of 1950) etc, for the Purpose of Creating a Sustainable Medical Insurance System (Law No.31 of 2015) 2016/April/1. [cited 2024 Oct. 31] Available from: https://laws.e-gov.go.jp/law/325AC0000000047

[19] Medical Division, Health Insurance Bureau, Ministry of Health, Labour and Welfare. List of Members of the Subcommittees and Committees in the Central Social Insurance Medical Council, documents at the 547th General Meeting (in Japanese). 2023/June/21. [cited 2024 Aug 31]. Available from: https://www.mhlw.go.jp/content/12404000/001110537.pdf

[20] Financial System Subcommittee, Fiscal System Council, Ministry of Finance. Minutes of the Meeting of the Subcommittee on Fiscal System. (in Japanese) 2024/April/16. [cited 2024 Aug 31]. Available from: https://www.mof.go.jp/about_mof/councils/fiscal_system_council/sub-of_fiscal_system/proceedings/proceedings/20240416zaiseia.html

[21] Pinho-Gomes AC, Stone J, Shaw T, et al. Values, principles, strategies, and frameworks underlying patient and public involvement in health technology assessment and guideline development: a scoping review. Int J Technol Assess Health Care. 2022;38(1): e46. 35655444 10.1017/S0266462322000289

[22] Mason RJ, Searle KM, Bombard Y, et al. Evaluation of the impact of patient involvement in health technology assessments: a scoping review. Int J Technol Assess Health Care. 2020;36(3):217–223. 32383420 10.1017/S0266462320000239

[23] Facey KM, Bedlington N, Berglas S, et al. Putting patients at the centre of healthcare: progress and challenges for health technology assessments. Patient. 2018;11(6):581–589. 30051315 10.1007/s40271-018-0325-5

[24] Moran R, Davidson P. An uneven spread: a review of public involvement in the National institute of health research’s health technology assessment program. Int J Technol Assess Health Care. 2011;27(4):343–347. 22004775 10.1017/S0266462311000559

[25] Gagnon MP, Dipankui MT, Poder TG, et al. Patient and public involvement in health technology assessment: update of a systematic review of international experiences. Int J Technol Assess Health Care. 2021;37: e36. 33541449 10.1017/S0266462321000064

[26] European Patient’s Forum. Patient involvement in health technology assessment in Europe: results of the EPF Survey. 2013. [cited 2024 Aug 31]. Available from: http://www.eupatient.eu/globalassets/projects/hta/hta-epf-final-report2013.pdf

[27] Hunter A, Facey K, Thomas V, et al. EUPATI guidance for patient involvement in medicines research and development. Health Technology Assessment. front Med (Lausanne). 2018 Sep 6; 5:231. 30238004 10.3389/fmed.2018.00231 PMC6136274

[28] Afzali H, Street J, Merlin T, Karnon J., The representation of public values in health technology assessment to inform funding decisions: The case of Australia’s national funding bodies. Int J Technol Assess Health Care. 2021;37: e22. 33455592 10.1017/S0266462320002238

[29] Livingstone H, Verdiel V, Crosbie H, et al. Evaluation of the impact of patient input in health technology assessments at NICE. Int J Technol Assess Health Care. 2021;37: e33. 33509314 10.1017/S0266462320002214

[30] Department of Health and Aged Care, Pharmaceutical Benefits Scheme. 2021. Fact sheet 4 - Strategic Agreement with Medicines Australia - Enhanced Consumer Engagement Process. 2021. [cited 2024 Aug 31]. Available from: https://www.pbs.gov.au/info/general/medicines-industry-strategic-agreement.

[31] Canada’s Drug Agency. Values for Patient Involvement in Action at CADTH. 2019. [cited 2024 Aug 31]. Available from: https://www.cda-amc.ca/cadth-framework-patient-engagement-health-technology-assessment-0

[32] National Institute for Health and Clinical Excellence. Patient and public involvement policy. [cited 2024 Aug 31]. https://www.nice.org.uk/about/nice-communities/nice-and-the-public/public-involvement/public-involvement-programme/patient-public-involvement-policy.

[33] Oortwijn W, Husereau D, Abelson J, et al. Designing and implementing deliberative processes for health eechnology assessment: A good practices report of a joint HTAi/ISPOR task force. Value Health. 2022; 25(6):869–886. 35667778 10.1016/j.jval.2022.03.018 PMC7613534

[34] Toledo-Chávarri A, Alvarez-Perez Y, Triñanes Y, et al. Toward a strategy to involve patients in health technology assessment in Spain. Int J Technol Assess Health Care. 2019;35(2):92–98. 30867077 10.1017/S0266462319000096

[35] Commonwealth of Australia, Department of Health and Aged Care, Pharmaceutical Benefits Scheme. Procedure guidance for listing medicines on the Pharmaceutical Benefits Scheme Version 2.5. 2022. [cited 2024 Aug 31]. Available from: https://www.pbs.gov.au/info/industry/listing/listing-steps.

[36] Canada’s Drug Agency. Reimbursement Reviews Process in Brief. 2020. [cited 2024 Aug 31]. Available from: https://www.cda-amc.ca/reimbursement-reviews-process-brief.

[37] Canada’s Drug and Health Technology Agency. Framework for Patient Engagement in Health Technology Assessment. 2019. [cited 2024 Aug 31]. Available from: https://www.cda-amc.ca/cadth-framework-patient-engagement-health-technology-assessment-0.

[38] National Institute for Health and Cera Excellence. Guide to the processes of technology appraisal. 2018. [cited 2024 Aug 31]. Available from: https://www.nice.org.uk/process/pmg19/resources/guide-to-the-processes-of-technology-appraisal-pdf-72286663351237. 27905710

[39] National Institute for Health and Clinical Excellence. NICE’s approach to public involvement in guidance and standards: a practical guide. 2015. [cited 2024 Aug 31]. Available from: https://www.nice.org.uk/media/default/About/NICE-Communities/Public-involvement/Public-involvement-programme/PIP-process-guide-apr-2015.pdf.

[40] EUnetHTA. HTA Core Model®. [cited 2024 Oct 31]. Available from: https://www.eunethta.eu/hta-core-model/.

[41] Low E, André-Vert J, Fanelli G, Galbraith M. International overview of health technology assessment training tools and materials for patients and consumers. Int J Technol Assess Health Care. 2023;39(1): e61. 37732420 10.1017/S0266462323000533 PMC11569957

[42] Rasburn M, Crosbie H, Tonkinson A, et al. Innovative patient involvement during Covid-19: Keeping patients at heart of HTA. Front Med Technol. 2021;3:793119. 35047972 10.3389/fmedt.2021.793119 PMC8757719

[43] Rasburn M, Livingstone H, Scott SE. Strengthening patient outcome evidence in health technology assessment: a coproduction approach. Int J Technol Assess Health Care. 2021; 37: e12. 33436125 10.1017/S0266462320002202

[44] Abelson J, Wagner F, DeJean D, et al. Public and patient involvement in health technology assessment: a framework for ACTION. Int J Technol Assess Health Care. 2016;32(4):256–264. 27670693 10.1017/S0266462316000362

[45] Tjeuw E, Wonder MJ. Analysis of consumer comments into PBAC decision-making (2014–9). [cited 2024 Aug 31]. Available from: 10.1017/S0266462321001744. 35115073

[46] Medical Division, Health Insurance Bureau, Ministry of Health, Labour and Welfare. Report from the organization specializing in cost-effectiveness evaluation, Agenda of the 596th Meeting of the General Committee Meeting in the Central Social Insurance Medical Council (in Japanese) 2024/October/9. [cited 2024 Oct. 31]. Available from: https://www.mhlw.go.jp/stf/newpage_44126.html.

[47] Medical Division, Health Insurance Bureau, Ministry of Health, Labour and Welfare. List of drugs and medical devices discussed in ’the Expert Committee of Cost-Effectiveness Evaluation’ in the Central Social Insurance Medical Council (in Japanese). [cited 2024 Aug 31]. Available from: https://www.mhlw.go.jp/stf/seisakunitsuite/bunya/0000121431_00302.html.

[48] Medical Division, Health Insurance Bureau, Ministry of Health, Labour and Welfare. Meeting information for the Special Committee of Cost Effectiveness Evaluation in the Central Social Insurance Medical Council (in Japanese). [cited 2024 Aug 31]. Available from: https://www.mhlw.go.jp/stf/shingi/shingi-chuo_128159.html

[49] Center for Outcomes Research and Economic Evaluation for Health, National Institute of Public Health. What we do. [cited 2024 Aug 31]. Available from: https://c2h.niph.go.jp/en/assessment/index.html

[50] Center for Outcomes Research and Economic Evaluation for Health, National Institute of Public Health. results. [cited 2024 Aug 31]. Available from: https://c2h.niph.go.jp/en/results/index.html

[51] Center for Outcomes Research and Economic Evaluation for Health, National Institute of Public Health. Academic Technology Assessment Group(ATAG) Reports [cited 2024 Dec 6]. Available from: https://c2h.niph.go.jp/results/atag.html

[52] Guidelines International Network. Beyond guidelines: tools to support patient involvement in health technology assessment, GIN Public Toolkit. 2021. [cited 2024 Aug 31]. Available from: https://g-i-n.net/toolkit/

[53] HTAi Patient and Citizen Involvement in HTA Interest Sub-Group2014. Completing A patient group submission template: Guidance for patient organisations. 2014. [cited 2024 Aug 31]. Available from: https://past.htai.org/wp-content/uploads/2018/02/PCISG-Resource-GuidanceandChecklist-Dec14.pdf.

[54] Cook N, Livingstone H, Dickson J, et al. Development of an international template to support patient submissions in health technology assessments. Int J Technol Assess Health Care. 2021;37(1):e50. 33789779 10.1017/S0266462321000167

[55] Staley K, Doherty C. It’s not evidence, it’s insight: bringing patients’ perspectives into health technology appraisal at NICE. Res Involv Engagem. 2016; 2:4. 29062505 10.1186/s40900-016-0018-y PMC5611625

[56] Berglas S, Jutai L, MacKean G, Weeks L. Patients’ perspectives can be integrated in health technology assessments: an exploratory analysis of CADTH common drug review. Res Involv Engagem. 2016; 2:21. 29062521 10.1186/s40900-016-0036-9 PMC5611639

[57] Wale J, Sullivan M. Exploration of the visibility of patient input in final recommendation documentation for three health technology assessment bodies. Int J Technol Assess Health Care. 2020; 36(3):197–203. 32375904 10.1017/S0266462320000240

[58] Canada’s Drug and Health Technology Agency. 2023. Procedures for CADTH Reimbursement Reviews. [cited 2024 Aug 31]. Available from: https://www.cadth.ca/cadth-procedures-reimbursement-reviews.

[59] Berglas S, Vautour N, Bell D. Creating a patient and community advisory committee at the Canadian agency for drugs and technologies in health. Int J Technol Assess Health Care. 2021;37: e19. 33478596 10.1017/S0266462320002251

[60] Medical Division, Health Insurance Bureau, Ministry of Health, Labour and Welfare. 2021. Minutes of the 59th Meeting of the Special Committee of the Cost Effectiveness Evaluation in the Central Social Insurance Medical Council (in Japanese). 2021/December/01. [cited 2024 Aug 31]. Available from: https://www.mhlw.go.jp/stf/shingi2/0000203149_00009.html.

[61] Saito S. How should we consider several factors other than cost-effectiveness analysis? Problems regarding appraisal (Comprehensive evaluation) Process J Pharmacoepidemiol, 2018; 23(1):29–39

[62] Medical Division, Health Insurance Bureau, Ministry of Health, Labour and Welfare. Main Issues Related to the Review of the Cost-effectiveness Evaluation System 3. Matters Related to Enhancing the Analytical System, Minutes of the 64th Meeting of the Special Committee of Cost Effectiveness Evaluation in the Central Social Insurance Medical Council. (in Japanese) 2023/September/13 [cited 2024 Oct 31]. Available from: https://www.mhlw.go.jp/stf/shingi2/0000182080_00015.html

[63] PPI Japan. EUPATI Japanese Table of Contents (in Japanese). [cited 2024 Aug 31]. Available from: https://www.ppijapan.org/eupati_toc

[64] EUPATI. 2016. Mini-course Starter Kit- Health Technology Assessment, EUPATI (in English). [cited 2024 Aug 31]. Available from: https://x.gd/FE8Sc

[65] Commonwealth of Australia, Department of Health and Aged Care. Co-design of an Enhanced Consumer Engagement Process for health technology assessment. [cited 2024 Aug 31]. Available from: https://www.health.gov.au/our-work/co-design-of-an-enhanced-consumer-engagement-process.

[66] Health and Global Policy Institute. 2024. Guidance on Patient and Public Involvement (PPI) in Health Policymaking: Necessary Initiatives and Good Examples from the Public and Government. [cited 2024 Aug 31]. Available from: https://hgpi.org/en/research/ncd-ppi-20240331.html?noredirect=en_US.

