# Supplementary material for: Value and Implementation of Patient and Public Involvement and Engagement in Health Technology Assessment for Japan: implications from systematic searches
Source: Int J Technol Assess Health Care. 2025 Feb 28;41(1):e14. doi: 10.1017/S026646232500008X (PMC11894397; doi:10.1017/S026646232500008X)
Supplement: Kaneyasu supplementary material [file S026646232500008Xsup001.pdf]

Supplementary Table 1:

Outline of literature review: not qualitative studies (conducted in single or multiple countries)

**Not-Q**	Presented	Umbrella/	**Each study**		Country			Particpants	PPIE purpose/value	PPIE implementation		Resource		Actions for	Information		**Serached in**	**reference #**
**study#**	in	Scoping	author	year	(in context)	Study type	Main theme			Stage of HTA	Form of involvement	Guideline	Education/training	patient/public input	from institution	feedback/evaluation	**Umbrella?**(author)	in main
1	Table1	U1	Pinho-Gomes	2022	Multinational	Scoping review	Summarize PPI principles, values, frameworks, and strategies in HTA and guideline development to evaluate impact of PPI	Patients and public	Representation, transparency, relevance, equity, fairness, and reconciling different types of knowledge.	Selecting and prioritizing topics, scoping, evidence review and analysis, drafting recommendations, dissemination, and evaluation of PPI	Individual or collective level: communication, consultation, and participation	Abelson (2016), EUPATI (2018), RedETS(2019), Perfetto (2018), CADTH (2021), HTAi (2022), INAHTA (2021)	de Wit (2019), CADTH & EUPATI: Support/training for patients and orgs Expertise and training for staff	de Wit (2019), CADTH (2021) & EUPATI (2018) Including the patient perspective	Gagnon (2015), Toledo - Chavarri (2019): Dissemination of HTA content to patients and health professionals.	de Wit (2019), CADTH & EUPATI: Feedback, review and evaluation of PPI	Pinho-Gomes AC(2022)	21
2	Table1	U2	Gagnon	2021	Multinational	Systematic review	Synthesize the barriers and facilitators of PPI in HTA and propose a framework to assess its impact	Patients and public	Awareness raising, relevance of PPI, perceptions of other stakeholders, development of patient materials, identification of collaborators	Different and each stage of HTA	Group or individual patients: participation, consultation or indirect participation	GRIPP checklist (2011/2017)	Organizational i.e. context: providing documents and training for patients in advance with adequate materials	Organizational culture: demonstrating openness toward patients' perspectives	Awareness raising: i.e. better information from government to patients and public about HTA	Decision-making context: providing feedback about their participation.	Gagnon(2021)	25
3	Table1	U3	Mason	2020	Multinational	Scoping review	Overview of methods for the evaluation of patient involvement impact	Patients	Patient Involvement provided insights into the technology under review that was not otherwise available.	From scoping topics, to interpreting evidence, and even drafting recommendations.	Patient group: direct involvement on HTA committees, and multiple forms	na	na	na	na	Combination of both qualitative and quantitative strategies may allow for the most comprehensive assessment of the impact of PI on HTA recommendations.	Mason(2020)	22
4	Table1	U1-2	Hunter	2018	Countries in European Union	Guidance document	Provide recommendations for activities to support patient involvement in HTA bodies and specific guidance for individual HTA processes	Patients	Relevance; Fairness; Equity; Legitimacy; Capacity building	Identifying and prioritaising. scoping, assessing, reviewing and disseminating	Group or individual: communication, consultation, and participation/ written submissions	HTAi/ISPOR materials, EUnetHTA core model, EUPATI guidance materials	HTA participants should receive training about involvement and consideration of patients perspectives Patients should have opportunity to receive mentoring and training about HTA	Should make systems for written submissions easy to use and appropriate support should be offered to individuals making submissions	Provide lay language versions of HTA outcome documents.	Patient involvement processes in HTA should be regularly reflected on and reviewed. Provide feedback to patients: how their submissions inform specific HTA.	Pinho-Gomes AC(2022)	27
5	Table1	S2	Oortwijn	2022	Multinational	Good practices report of a joint HTAi/ISPOR task force	Guidance and checklist development for deliberative processes for HTA	Patients, public/citizens, and other stakeholders	Process indicators (e.g., transparency, impartiality, inclusivity, timeliness consistency and verifiability), outcome indicators (e.g., Increase of public trust, and acceptance of decisions)	Identification and prioritization of relevant topics for HTA, providing scientific advice, scoping, assessment, and synthesis of relevant information, contextualization of HTA, development and communication of the output(s), monitoring and evaluation	No info about group or individual: Face-to-face, virtual, written	Stakeholder participation checklist (Jansen 2018), HTAi patient group submission template (2014), Good practices report of a joint HTAi/ISPOR task force (2022)	Training and education should be considered so that individuals can participate fully in an informed deliberation	Poorly implemented deliberative processes that do not manage these power dynamics can lead to distractions that dilute fruitful discussion.<br>This creates ethical challenges for patients as well as risks of delay or poor decisionmaking.	Recording (video, audio, or transcript), written report (e.g., minutes)	Monitoring and evaluating a deliberative process involving questionnaires, interviews, document reviews, or live meetings held with participants	Searched in scoping review	33
6	not in table	S1	Low	2023	France(HAS)*	Literature search, website review, semi-structured interview	Training material development (overveiw of HTA materials among countries)	Patients and consumers	na	training	na	HAS(2021),HIQA(2016), HTW(2019), NICE,(2021), SHTG(2021), SMC(2017), ECPC(2021), ANSM(2021)	1.Understanding HTA and the role of HAS, 2. PPI in HTA at HAS	na	na	na	Low(2023)	41
7	not in table	U1-3	Toledo-Chavarri,	2019	Spain(RedETS)*	Systematic search, a qualitative study, a Delphi consultation	Develop a feasible and effective strategy	Patients	Values: relevance, justice, equity, legitimacy, and capacity building / objectives: democratic, legitimacy, instrumental, and scientific	Identification and prioritization, setting objectives and scope of assessment, evidence review, elaboration of recommendations, review and presentation of allegations, dissemination	Individual/group: surveys, stakeholder meetings, Interviews/focal groups, Participation in the expert panel anr/or citizens panel, public consultation	G-I-N PUBLIC Toolkit, Participación de los pacientes en la Evaluación de Tecnologías Sanitarias: manual metodológico.	Including the patients need for informative and formative actions on HTA, the training of HTA professionals in PI techniques,	na	Increased dissemination of results with patient friendly versions.	na	Pinho-Gomes AC(2022)	34
8	not in table	U2-1	Abelson	2016	Canada(Ontarios)	Synthesis of international practice and published literature, a dialogue with local, national and international stakeholders	PPI framawork for OHTAC(Canada)	Public and patients	Goal: instrumental goal(quality inprovement), development goal(raising awareness of HTA), Value: incorporating societal and/or patient perspectives and/or values at stake.	Various stages in the HTA process(Scoping, analysis, draft recommendation, professional public consultation)	Individual/ group: public reviews of draft documents, surveys, face-to-face discussions (that occur most typically at the appraisal and draft recommendation stage)	Public Engagement for Health Technology Assessment at Health Quality Ontario—Final Report From the OHTAC Public Engagement Subcommittee(2015)	(i) Guiding principles and goals of PPI in HTA; (ii) establishment of a common language to support PPI;(iii) a flexible array of approaches; (iv) ongoing evaluation of PPI to drive improvement.	Supportive organizational culture, appropriate supports for patient/public committee members and those interact ing with them, dedicated time devoted to patient perspectives on meeting agendas, and strategic use of new and existing networks of patient organizations for external consultations	Multimadia dissemination, plain language summary, targeted dissemination to high priority group	Focus on the basic formative evaluation metrics to determine whether the intended goals of the PPI activities are being achieved.	Pinho-Gomes AC(2022),Gagnon(2021)	44
9	not in table	S4	Rasburn	2021a	UK(NICE)	(Document) descriptive analyses.	Virtual engagement support during NICE's rapid shift to virtual meetings	Patients	na	Virtual committee meetings, developing guidance (case study)	No info about group or individual: participation	na	Provided technical training before meetings to ensure participants can use the software and live technical support.	(1) Accessibility<br>(2) inclusivity<br>(3) transparency<br>(4) intrapersonal relationships and committee dynamics.	na	Evaluation and analyses for the impact on PPI and patient contributors and introduced measures to mitigate risk of exclusion and avoid tokenistic involvement.	Searched in scoping review	42
10	not in table	S3	Tjeuw	2022	Australia(PBAC)	(Document) descriptive analyses.	Analyze and review consumer comments in the PBACs decision-making process	Consumers	na	Consumer input on the assessment/ draft outcome, final meeting. Consumer representation on decision-making committee.	Individual/ group: comments, sponser hearings, consumer hearings, consumer issues are highlighted by the consumer representatives during discussion of agenda items	PBAC, NICE, **Institute for Clinical and Economic Review,** and CADTH	na	Notify the relevant patient group/s that the agenda for an upcoming meeting has been published and that there<br>is at least one agenda item of interest to them.<br>Advise the relevant patient group/s about some non commercially sensitive aspects of its submission<br>Assist the relevant patient group/s in the preparation of comments to the PBAC<br>Fund relevant patient group/s to undertake communication and advocacy activities.	na	na	Searched in scoping review	45
11	not in table	U3-1	Berglas	2016	Canada(CADTH)	Document review	Explored whether, and how, patient insights were integrated into assessment reports	Patients	Better understanding of the potential value of a new medicine from a patient perspective.	na	na	CADTH. Patient input templates. Procedure for the CADTH Common Drug Review.	na	CADTH reviewers then (after patient group confirmation for the CADTH draft version) translate patient groups' descriptions of specific needs that are not met with current treatment, or their hopes regarding new therapies, into outcomes used within the relevant assessment protocols.	na	Since June 2014, CADTH has held additional training sessions for clinical reviewers and Canadian Drug Expert Committee, and introduced feedback letters to patient groups.	Mason(2020)	50
12	not in table	S6	Rasburn	2021b	United Kingdom (NICE)	Process report	Review NICE HTA PPI methods and processes and coproduce recommendations	Patients	(PPI is not tokenistic but adds value)	Strategy to recommendations	Patient organization representatives: coproduction working group	Public Involvement Programme@NICE	Providing training and support for participants to increase their understanding of the HTA process and then submitting evidence	Provide tailored support and structured training to help participants navigate and understand HTA documents and processes.	na	Provide feedback to patient stakeholders explaining how their evidence submission influenced the HTA.	Searched in scoping review	43

Outline of literature review: not qualitative studies (conducted in single or multiple countries)

**Not-Q**	Presented	Umbrella/	**Each study**		Country			Particpants	PPIE purpose/value	PPIE implementation		Resource		Actions for	Information		**Serached in**	**reference #**
**study#**	in	Scoping	author	year	(in context)	Study type	Main theme			Stage of HTA	Form of involvement	Guideline	Education/training	patient/public input	from institution	feedback/evaluation	**Umbrella?**(author)	in main
**13**	not in table	S7	Berglas	2020	Canada(CADTH)	Process report	Create patient and community advisory Committee.	Patients and public	na	Across all CADTH programs	Individuals or organizations as members: patient and community advisory committee	CADTH 2018-2021 Strategic Plan	Patient and Community Advisory Committee Members help CADTH build cultural competence (identifying bias and improving communication) .	na	na	Public and patient engagement evaluation tool@McMaster University [https://ppe.mcmaster.ca/resources/public-and-patient-engagement-evaluation-tool/] will be used.	Searched in scoping review	53
**14**	not in table	S6	Livingstone	2021	United Kingdom (NICE)	Survey	Assess the level and the type of impact of patient input to highly specialised technologies	patient	Patient input helped interpret other evidence by providing context	Scoping, evidence submission, committee meeting, consultation, final recommendation, publication, review[suppl]	Individual/group: particilation, presentation, comments, appearl, review [suppl]	Values and Standards for Patient Involvement in HTA, by HTAi	na	na	na	Feedback is given to patient advocacy groups who have contributed to an HTA, to share what contributions were most helpful and provide suggestions to assist their future involvement.	Searched in scoping review	29
**15**	not in table	S8	Afzai	2021	Australia(PBAC)	Survey	How to reorient HTA to reflect public priorities	public	Ensure the legitimacy and fairness of public funding decisions	Comparative safety, economic evaluation, other relevamt factors	Individual/ group: participation, patient input for consultation	Co-design of an Enhanced Consumer Engagement Process | Australian Government Department of Health and Aged Care	na	na	na	na	Searched in scoping review	28
**16**	not in table	U3-2	European Patient's Forum	2013	Multinational	Survey	Identify the current situation, informing and building the capacity of patient organisations,	patient	Patient organisations are sceptical about the impact of PPI perceived by the HTA agencies.	Production of information, internal review, identification, filtration, and prioritisation.	Patient group: public consultations, in providing patient evidence and in appeals against decisions/ final reccomendations	EUPATI guidance on patient involvement with HTA(Hunter 2018)	Capacity building (organisational development, workforce development, partnership working, leadership and resources allocation)	Better and timely communication with patient organisations	Not in detail	The impact of patient involvement should be assessed in a structured way.	Mason(2020)	26

U-1 to U-3 are derived from primary screenings; U1-x means selected from the U1 reference, while S-1 to S-10 mean extracted from the scoping review.

*They do not strictly meet the criteria for a scoping review (information from target countries and relevant organisations). However, as the reports were comprehensive and user-friendly summaries of relevant information, they were retained in the literature set.

Abbreviations

ANSM:Agence Nationale de sécurité du médicament et des produits de santé; CADTH: Canadian Agency for Drugs and Technologies in Health; ECPC: European Cancer Patient Coalition; EUnetHTA: European Network for Health Technology Assessment; EUPATI: European Patients’ Academy on Therapeutic Innovation; GRIPP: Guidance for Reporting Involvement of Patients and the Public; HAS: French National Authority for Health; HIQA: Health Information and Quality Authority; HTA: health technology assessment; HTAi: HTA International; HTW: Health Technology Wales; INAHTA: International Network of Agencies for Health Technology Assessment, ISPOR: International Society for Pharmacoeconomics and Outcomes Research; na: not applicable; NICE: National Institute of Health; and Care Excellence; OHTAC: Ontario Health Technology Advisory Committee; orgs: organizations; PBAC: Pharmaceutical Benefits Advisory Committee; PPI: patient and public involvement, PPIE: patient and public involvement and engagement; RedETS: Spanish Network of Health Technology Assessment; SHTG: Scottish Health Technologies Group; SMC: Scottish Medicines Consortium, Healthcare Improvement Scotland

Supplementary Table 2.
Outline of literature review: qualitative studies

Quality study#	Umbrella/ scoping	**Each study** author	year	Country (in context)	Study type	Main theme	Particpants	PPIE purpose/value	PPIE implementation Stage of HTA	Form of involvement	Resource Guideline	Education/training	Actions for patient/public input	Information from institution	feedback/evaluation	**Serached in umbrella?**(author)
1	U3-2	Abelson	2013	Canada(Ontarios)	Descriptive qualitative analysis	Tracing the impacts of a deliberative participatory structure (the Citizens' Reference Panel on Health Technologies)	Citizens	Macro-level: raising awareness micro-level: informing recommendations	Vignette(seek input on the outcome to be assessed), evidence-based process, draft analysis and recommendation, public engagement evaluation, post-recommendation phase	Monthly meeting with cirizen panel and the expert advisory committee	Public Engagement Subcommittee of the Ontario Health Technology Advisory Committee. Final report (2015)	na	Overview of each discussion topic followed by a Q&A session and a combination of large and small group discussions with reporting back and thematic summarizing sessions.	Background materials: (HTA evidence summaries and draft recommendations, relevant review articles and newspaper clippings, and a workbook)circulated in advance of each meeting	na	Pinho-Gomes AC(2022), Gagnon(2021)
2	U3-3	Brereton	2017a	Multinational	Stakeholder consultation or a qualitative research design	Report on stakeholder involvement in the Integrated health technology assessment for the evaluation of complex technologies(INTEGRATE-HTA) project [palliative care]	Lay members (e.g. patients, carers, family members and members of public organisations or groups) and professionals	key shared ensure priorities are addressed and research findings translated into practice. (Potentially increases the value of the HTA findings.)	Scoping, evidence collection and assessment, applicability, decision making	Depend on the countries	The five-step INTEGRATE-HTA Model (2016)	na	Depend on the countries	na	PiiAF: Public Involvement Impact Assessment Framework (Popay 2014)	Gagnon(2021)
3		Brereton	2017b													
4	U3-4	Boothe	2019	Canada(Ontatio, CADTH)	Interview	Describes goals of PPI with primarily related to legitimate processes (legitimacy goals) or improved decisions (instrumental goals)	Patient group representatives and lay (public or patient) members	"Improve the legitimacy and achieve "fair and transparent processes," and "to inform policy decisions" and to find "meaningful ways to gather input from relevant patient and publics and make better quality decisions."	Regardless of the stage	Any form implemented	na	na	na	na	Feedback letter to patient group	Gagnon(2021)
5	U3-5	Cockcroft	2019	United Kingdom	Qualitative study accompanied with a case	Understand public knowledge/experience sharing during meetings with researchers	Public and patients	Direct lived personal experience; learnt knowledge; and the experience and values of others	three group-based public involvement meetings and one community drop-in session	Participation	INVOLVE. Briefing Notes for Researchers: Involving the public in NHS(2012)	na	Group-based involvement meetings allow for the synergistic combination of individual knowledge and experience	na	PiiAF: Public Involvement Impact Assessment Framework (Popay 2014)	Gagnon(2021)
6	U3-6	Pomey	2019	Canada (Institut national d' excellence en sant é et en services sociaux: INESSS)	Observation and interview	Describe and propose the co-construction process recommendations with best practices	Patients	Create more patient-focused guidance	Review of literature, validation of contens, recommendations	Expert patient committee, co-constraction committee	INESSS, Azzi(2018)	a lack of training in PI on the part of the scientific team before this project.	Recruitment of patients for the expert patient committee employed a methodology developed by Pomey and that recommends 4 steps.	na	na	Gagnon(2021)
7	U4-3	Staley and	2016	United Kingdom (NICE)	Semi-structured interviews	How the written patient statement adds value to the process	Patients	Aid committees interpretation and understanding of the evidence	Initial scoping stage to the appraisal.	na	NICE documents(2015), INVOLVE(2015)	na	na	na	na	Mason(2020)
8	S8	Boothe	2021	Canada	Comparative qualitative analysis (held in 2014, 2016, 2018, and 2019)	Examines the adoption (legitimacy) of PPI in pharmaceutical assessment among expert in Canada.	Patients and public	Scientific legitimacy/ democratic legitimacy	Focused on technical committee	Committee participation	na	na	na	na	na	Searched in scoping review
9	S9	Mercer	2020	Canada (CADTH)	Qualitative interview study	Explores experiences and perceptions among patient groups participating in CADTH/pan-Canadian Oncology Drug Review (pCODR) process	Patients	Bringing the patient voice to decision making tables	Participating in the CADTH's pan-Canadian Oncology Drug Review (pCODR) process	Input at the start of a review and feedback on the initial recommendation (basically by patient group)	EUPATI (2018)	Training in HTA may not be sufficient for the patient groups to provide informative input to the pCODR Expert Review Committee deliberations.	na	na	na	Searched in scoping review

U1-x means extracted from the umbrella review (i.e. selected from the U1 reference), while S-1 to S-9 mean extracted from the scoping review.

Reference

1 Abelson J, et al. Assessing the impacts of citizen deliberations on the health technology process. Int J Technol Assess Health Care. 2013;29(3):282-9.
2 Brereton L, et al. STAKEHOLDER INVOLVEMENT THROUGHOUT HEALTH TECHNOLOGY ASSESSMENT: AN EXAMPLE FROM PALLIATIVE CARE. Int J Technol Assess Health Care. 2017;33(5):552-561
3 Brereton L, et al. Lay and professional stakeholder involvement in scoping palliative care issues: Methods used in seven European countries. Palliat Med. 2017;31(2):181-192
4 Boothe K. "Getting to the Table": Changing Ideas about Public and Patient Involvement in Canadian Drug Assessment. J Health Polit Policy Law. 2019;44(4):631-663.
5 Cockcroft EJ, et al. How is knowledge shared in Public involvement? A qualitative study of involvement in a health technology assessment. Health Expect. 2020;23(2):348-357.
6 Pomey MP, et al. Co-construction of health technology assessment recommendations with patients: An example with cardiac defibrillator replacement. Health Expect. 2020;23(1):182-192
7 Staley K, et al. It's not evidence, it's insight: bringing patients' perspectives into health technology appraisal at NICE. Res Involv Engagem. 2016:2:4.
8 Boothe K. (Re)defining legitimacy in Canadian drug assessment policy? Comparing ideas over time. Health Econ Policy Law. 2021;16(4):424-439
9 Mercer RE, et al. Are We Making a Difference? A Qualitative Study of Patient Engagement at the pan-Canadian Oncology Drug Review: Perspectives of Patient Groups. Value Health. 2020;23(9):1157-1162

Abbreviations

CADTH: Canadian Agency for Drugs and Technologies in Health; EUPATI: European Patients' Academy on Therapeutic Innovation; HTA: health technology assessment; na: not applicable; NICE: National Institute of Health; and Care Excellence; OHTAC: Ontario Health Technology Advisory Committee; PBAC: Pharmaceutical Benefits Advisory Committee; PPI: patient and public involvement, PPIE: patient and public involvement and engagement;
